# Nicholas of Russia

**Published:** 1860-04

**Authors:** 


### NICHOLAS OF RUSSIA.

A PRIVATE memoir of the late Emperor Nicholas the First was published and distributed among the more immediate relatives and friends of the autocrat of sixty millions of human beings. It is made up from his private papers, from official records and other authentic sources; hence it may be regarded as a true representation of the inner life of the real character of that distinguished personage. From a perusal of it, by the courtesy of a friend, who was in St. Petersburgh at the time of the Emperor's death, and whose personal knowledge confirms, as far as it goes, the literal truth of the memoir, we are constrained to say, that perhaps not one in a million of all who speak the English language has a proper estimate of the late Czar. As an emperor, a husband, a father, a brother, a master, a friend, there is a beauty in his character which has few parallels among sovereigns. No one can rise from its perusal without a deep impression that he was a Christian. A synopsis of the memoir will be found in the April number of the FIRESIDE MONTHLY.

